# The Participation of HPV-Vaccinated Women in a National Cervical Screening Program: Population-Based Cohort Study

**DOI:** 10.1371/journal.pone.0134185

**Published:** 2015-07-28

**Authors:** Eva Herweijer, Adina L. Feldman, Alexander Ploner, Lisen Arnheim-Dahlström, Ingrid Uhnoo, Eva Netterlid, Joakim Dillner, Pär Sparén, Karin Sundström

**Affiliations:** 1 Department of Medical Epidemiology and Biostatistics, Karolinska Institutet, Stockholm, Sweden; 2 Medical Research Council Epidemiology Unit, University of Cambridge, Cambridge, United Kingdom; 3 Public Health Agency of Sweden, Stockholm, Sweden; 4 Department of Clinical Sciences, Faculty of Medicine, Lund University, Malmö, Sweden; 5 Department of Occupational and Environmental Dermatology, Skåne University Hospital, Malmö, Sweden; 6 Department of Laboratory Medicine, Karolinska Institutet, Stockholm, Sweden; ISPO, ITALY

## Abstract

**Background:**

Concerns have been raised that HPV-vaccination might affect women’s cervical screening behavior. We therefore investigated the association between opportunistic HPV-vaccination and attendance after invitation to cervical screening.

**Methods:**

A cohort of all women resident in Sweden, born 1977-1987 (N=629,703), and invited to cervical screening, was followed October 2006 - December 2012. Invitations to screening were identified via the National Quality Register for Cervical Cancer Prevention, as was the primary outcome of a registered smear. Vaccination status was obtained from two nationwide health data registers. Hazard ratios (HR) were estimated using Cox regression adjusted for age, education level and income (HR_adj_). Women were individually followed for up to 6 years, of which the first and second screening rounds were analyzed separately.

**Results:**

Screening attendance after three years of follow-up was 86% in vaccinated women (N=4,897) and 75% in unvaccinated women (N=625,804). The crude HR of screening attendance in vaccinated vs. unvaccinated women was 1.31 (95% CI 1.27-1.35) in the first screening round. Adjustment for education and income reduced but did not erase this difference (HR_adj_=1.09, 95% CI 1.05-1.13). In the second screening round, attendance was likewise higher in HPV-vaccinated women (crude HR=1.26, 95% CI 1.21-1.32; HR_adj_=1.15, 95% CI 1.10-1.20).

**Conclusions:**

HPV-vaccination is so far associated with equal or higher attendance to cervical screening in Sweden in a cohort of opportunistically vaccinated young women. Most but not all of the difference in attendance was explained by socioeconomic differences between vaccinated and unvaccinated women. HPV vaccine effectiveness studies should consider screening attendance of HPV-vaccinated women when assessing incidence of screen-detected cervical lesions.

## Introduction

Human papillomavirus (HPV) vaccination was introduced in Sweden in October 2006. The quadrivalent HPV vaccine (qHPV) was approved in September 2006 and the bivalent HPV vaccine (bHPV) in September 2007. A nation-wide opportunistic HPV vaccination programme was in effect during 2007–2011 and offered a 50% subsidy towards the cost for each dose prescribed to girls aged 13–17 years [1–2]. The vaccines were also available for women >18 years, but at full expense for the three doses (~280 euro for bHPV and 330 euro for qHPV in 2010) [3]. In 2012, a nation-wide organized school-based vaccination programme targeting 10–12 year old girls and a nation-wide catch-up programme was organized for girls 13–18 years [4].

The organized Swedish cervical screening programme issues invitations three years after the latest smear for women aged 23–50 and every five years for women aged 51–60. Repeated annual invitations to screening are issued by regional offices to all women in the population register who have not been screened according to recommendations [5]. The overall coverage proportion of women that have been screened at least once within the preceding recommended screening interval is 80% [6].

HPV vaccination is not therapeutic against prevalent HPV infections [7], and if vaccinated after sexual debut, females may already be exposed to the vaccine HPV types [8]. Also, current vaccines do not cover all high-risk HPV types [9]. Continued adherence to screening programmes is thus necessary for HPV-vaccinated women to ensure adequate cervical cancer protection, and to provide a basis for population-wide evaluation of protective effects against cervical cancer precursors. Since the introduction of HPV vaccination, there has however been a concern that HPV-vaccinated girls and women might erroneously feel fully protected against cervical cancer and therefore reduce their screening attendance [10].

A study in Australian university students found that knowledge of cervical screening guidelines was generally poor, but that screening uptake did not differ between vaccinated and unvaccinated women [11]. In contrast, another Australian study on women age 18–27 found that 19% viewed screening as being of low importance following HPV vaccination [12] and several studies have found poor understanding for the function and need of screening in vaccinated girls in, among other countries, the United Kingdom [13–14]. It is clearly essential to assess whether these attitudes manifest in differences in actual screening behaviour. As part of the Public Health Agency of Sweden’s plan for monitoring HPV vaccination, we performed a nation-wide cohort study of cervical screening attendance among opportunistically HPV-vaccinated young women in Sweden.

## Methods

### Study population

The population in this nationwide study comprised an open cohort of all women born 1977–1987, resident in Sweden and invited to cervical screening during the study period. The first issued invitation to screening after October 1^st^, 2006 defined study entrance. Women were followed until they attended screening, emigrated, died, or the study period ended on December 31^st^, 2012—whichever occurred first. Women who emigrated or died before the start of follow up, or had no record of screening invitation, were excluded.

### Data collection

Data were collected using the Swedish nationwide population-based health registers. All women born between 1977 and 1987 were selected from the Total Population Register and were linked to the registers described below, using the unique personal identification number (PIN) [15]. Information on deaths was retrieved from the Causes of Death Register and information on migration from the Migration Register. The Database for Health Insurance and Labour Market Studies, the Education Register, and the Multi-Generation Register, were used to obtain information on education level and income of study participants and their parents at the start of follow-up (i.e. at study entry). Data on all invitations to screening and on all smears (both opportunistic and organized), as well as cervical histo-pathologies were retrieved from the National Cervical Screening Registry (NKCx), which is 100% complete for Sweden since 1995 [6].

The Swedish HPV Vaccination Register (SVEVAC) and Prescribed Drug Register (PDR) were used to obtain women’s HPV vaccination status and date of vaccination.

SVEVAC is maintained by the Swedish Association of Local Authorities and Regions, and holds information on qHPV and bHPV administrations and was launched in parallel with the start of HPV vaccination [16]. Participation during the study period included informed consent to participate in register linkage studies. The coverage in 2012 compared to number of sold doses in the nation was 80–85% for the years 2006–2010 [1] and 92% for the year 2012, with a time lag of 3 months. PDR is an automated register including dispensed vaccine prescriptions for subsidized vaccination in girls age 13–17 years, and was used as a complement for both bHPV and qHPV data. The PDR is 100% complete since July 2005 [17].

### Statistical analysis

A descriptive analysis on HPV vaccination and socio-demographic status was conducted among women who were eligible for cervical screening but with no record of invitation. In the cohort of women who were invited to screening, the attendance among HPV-vaccinated and unvaccinated women, respectively, was calculated as cumulative incidence proportions over time from invitation. Cox regression was used to estimate hazard ratios (HR) with corresponding 95% confidence intervals (CI) of cervical screening attendance among HPV-vaccinated versus unvaccinated women, with attained age as the underlying time scale. HPV vaccination status was handled as a time-varying exposure. Women were considered to be HPV-vaccinated if they received at least one vaccine dose during the study period for the primary analyses.

Attendance was measured over the entire study period, and in the first and second screening round, respectively—the latter to examine potential variations in screening behaviour over time. The first round of screening was defined as attendance during 0–3 years after first invitation during the study period.

Entry to the second screening round was defined as whichever occurred first: the first issued invitation after attendance to screening during follow up (i.e. after three years in women who were screened normal), or the first issued invitation to screening after three years of non-attendance. Finally, we investigated attendance in the second repeated screening round, in the subgroup of women who attended in the first.

The crude main effects model was extended with additional co-variates based on explorative modelling. Adjustments were made for education level and individual income. Education level was categorized into missing; less than high school; high school, and university studies. Women with a missing education level were retained as a separate category, as the women in the study cohort were very young. Furthermore, an association between increasing education level and likelihood of getting vaccinated has been shown in Sweden [1]. The impact of education was therefore further assessed in an interaction model by including an interaction term between vaccination status and education level. There was moderate but not conclusive evidence for improvement in fit by including the interaction term. Results from both the main effects and interaction models are therefore reported. Individual income was categorized in quartiles based on the entire cohort, where women with no disposable income recorded were removed since this did not improve model fit. Adjustments for parental education, household income, and county were also explored, but this did not result in a better model fit. These variables were thus not included in the final model. Model comparative goodness-of-fit was assessed using the Akaike information criterion (AIC) [18]. A graphical representation of the scaled Schoenfeld residuals was used to check for violations of the proportional hazards assumption [19]. All tests of statistical significance were two-sided and p<0.05 was considered statistically significant.

### Sensitivity analyses

In separate sensitivity analyses, women were considered to be HPV-vaccinated only after the full 3 doses. Additional analyses exploring alternate definitions of the first screening round were performed using cut-offs of 2.5, 3.5, and 4 years instead of 3 years. Finally, an analysis was performed including calendar year of study period (2006–2012) as a covariate, to investigate whether the associations varied by study year.

Data management was done with SAS statistical software version 9.4 (SAS Institute Inc., Cary, NC). Statistical analyses were performed with Stata 13 (StataCorp LP, College Station, TX). The study was approved by the Regional Ethical Review Board in Stockholm, Sweden (No. 2012/216-32 and 278/2012-4.1.2) which determined that written informed consent by the study participants was not required.

## Results

### Study population

**Fig 1:**
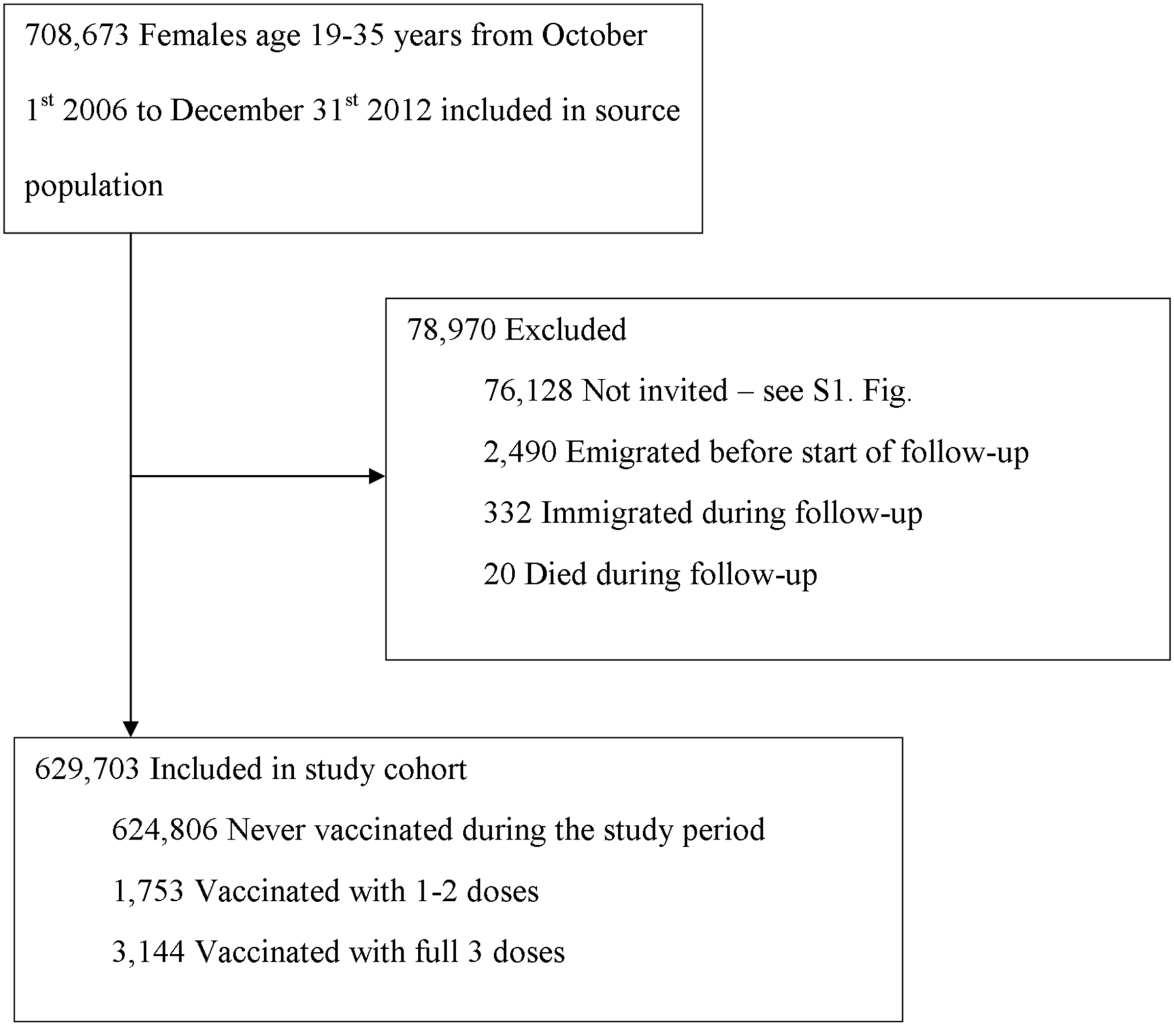
Details on study exclusions and population analyzed to study the association between HPV vaccination status and attendance to cervical screening after invitation.

**Fig 2:**
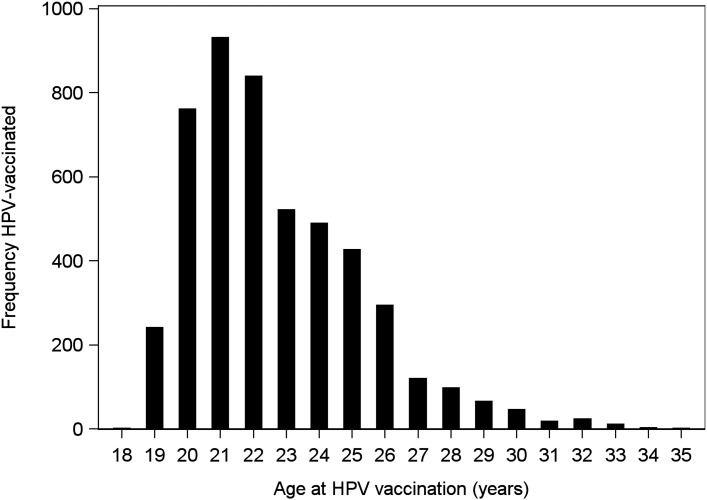
Age at opportunistic HPV vaccination among HPV-vaccinated females in the study cohort.

A total of 708,673 women born 1977–1987 were included in the study population (Fig 1). Of these, 76,128 women were excluded due to having no record of invitation during the study period. The main reasons for this were migration or having taken an opportunistic cervical smear without invitation (S1 Fig). Among the 25,860 women who were not included in the final study cohort since they already had an opportunistic smear, 2.2% were HPV-vaccinated. In total, the lack of invitation remained unexplained for only 3,670 women (0.2% of which were HPV-vaccinated). Out of these, 2,195 women (60%) lacked information on other register data such as birth country, indicating suboptimal register information which likely explains the lack of invitation during the study period. In the final study cohort of 629,703 women, 4,897 (0.8%) were HPV-vaccinated with at least one dose and were followed for an average of 1.44 (standard deviation 1.67) years. Individual incomes ranged from 0–23,576 Euro/year. HPV-vaccinated women were more likely to have university level studies and the highest income quartile (Table 1). HPV vaccination was most commonly done at age 20–22 years (Fig 2).

**Table 1:** Number of individuals, events, and person time by HPV vaccination status in the entire cohort and per education and income level.

	HPV-vaccinated with ≥1 dose^a^			Unvaccinated		
	Individuals (n, %)	Attended screening (n)	Person time (years)	Individuals (n, %)	Attended screening (n)	Person time (years)
Cohort total	4,897 (100)	3,919	5,156	625,804 (100)	490,781	900,686
Education						
*Missing data on edu*	83 (1.7)	36	104	54,302 (8.7)	26,182	102,368
*Less than high school*	108 (2.2)	73	148	59,540 (9.5)	41,429	118,860
*High school*	1,767 (36.1)	1,419	1,791	251,548 (40.2)	204,235	354,800
*University*	2,939 (60.0)	2,391	3,113	260,414 (41.6)	218,935	324,658
Income						
*1* ^*st*^ *quartile*	653 (13.4)	476	669	154,091 (25.1)	100,858	283,950
*2* ^*nd*^ *quartile*	1,040 (21.3)	836	1,135	153,406 (25.0)	124,137	222,143
*3* ^*d*^ *quartile*	1,444 (29.6)	1,183	1,451	153,240 (25.0)	127,866	206,725
*4* ^*th*^ *quartile*	1,748 (35.8)	1,422	1,889	152,711 (24.9)	132,584	178,353

^a^ Women were HPV-vaccinated with at least 1 dose.

### Screening attendance

**Fig 3:**
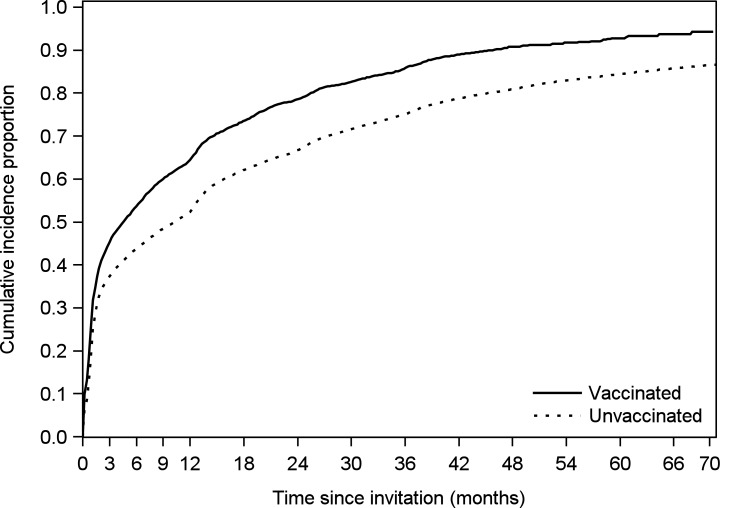
Cumulative incidence proportions of screening attendance since first invitation to screening by vaccination status.

HPV-vaccinated women had a higher cumulative incidence proportion of screening attendance than unvaccinated women, an association that was established immediately after invitation. After three years, 86% of HPV-vaccinated women had attended screening, compared to 75% of unvaccinated women (log rank test for curve: *P*<0.0001). Attendance remained higher in the HPV-vaccinated group throughout follow-up (Fig 3).

When stratifying on education level, attendance was higher among HPV-vaccinated women in all groups (S2 Fig).

In the regression analysis based on attained age; the overall crude HR of screening attendance was 1.28 (95% CI 1.24–1.32) for vaccinated compared to unvaccinated women. When adjusting for income and education, the adjusted HR (HR_adj_) for the entire study period was 1.05 (95% CI 1.02–1.08) in the main effects model (Table 2). In the analysis by rounds, screening attendance in those vaccinated was higher in both the first round and in the second round, irrespective of income or education (Table 2).

**Table 2:** Crude and adjusted hazard ratios from the main effects model of screening attendance in HPV-vaccinated women compared to unvaccinated women during the entire study period, and by round 1 and 2 during follow-up.

	Attendance over entire study period			
	Crude HR (95% CI) ^a^	*P* value	HR_adj_ ≥1 dose (95% CI)^b^	*P* value
**Unvaccinated**	Ref.		Ref.	
**HPV-vaccinated**	1.28 (1.24–1.32)	*<0*.*0001*	1.05 (1.02–1.08)	*0*.*004*
	**Attendance to screening round 1**			
**Unvaccinated**	Ref.		Ref.	
**HPV-vaccinated**	1.31 (1.27–1.35)	*<0*.*0001*	1.09 (1.05–1.13)	*<0*.*0001*
	**Attendance to screening round 2**			
**Unvaccinated**	Ref.		Ref.	
**HPV-vaccinated**	1.26 (1.21–1.32)	*<0*.*0001*	1.15 (1.10–1.20)	*<0*.*0001*

^a^ Unadjusted hazard ratios (HRs) with corresponding confidence intervals (CIs).

^b^ HRs with corresponding CIs adjusted for income and education. Women were HPV-vaccinated with at least 1 dose.

The AIC value for the interaction model was decreased by 7, indicating moderate evidence for improvement in fit by including the interaction term. Screening attendance by education level from this model is therefore also reported (Table 3). In this analysis, HPV-vaccinated women of missing, lower than high school, high school, or university education level were at least as likely as unvaccinated women to attend screening over the entire study period, and by rounds, respectively (Table 3).

**Table 3:** Adjusted hazard ratios of screening attendance by HPV vaccination status and education level during the entire study period, and by round 1 and 2 during follow-up.

	Attendance over entire study period		Attendance to screening round 1		Attendance to screening round 2	
	HR_adj_ ≥1 dose (95% CI)^a^	*P* value	HR_adj_ ≥1 dose (95% CI)^a^	*P* value	HR_adj_ ≥1 dose (95% CI)^a^	*P* value
**Missing data on education**						
Unvaccinated	Ref.		Ref.		Ref.	
Vaccinated	1.31 (0.95–1.82)	*0*.*105*	1.56 (1.12–2.18)	*0*.*008*	1.87 (0.70–4.99)	*0*.*210*
**< High school**						
Unvaccinated	Ref.		Ref.		Ref.	
Vaccinated	1.23 (0.98–1.55)	*0*.*079*	1.37 (1.08–1.74)	*0*.*009*	1.19 (0.84–1.68)	*0*.*335*
**High school**						
Unvaccinated	Ref.		Ref.		Ref.	
Vaccinated	1.12 (1.06–1.18)	*<0*.*0001*	1.14 (1.08–1.20)	*<0*.*0001*	1.21 (1.10–1.33)	*<0*.*0001*
**University studies**						
Unvaccinated	Ref.		Ref.		Ref.	
Vaccinated	1.00 (0.96–1.05)	*0*.*850*	1.05 (1.01–1.09)	*0*.*026*	1.13 (1.08–1.19)	*<0*.*0001*

^a^ Hazard ratios (HRs) with corresponding confidence intervals (CIs) adjusted for income and including an interaction term between vaccination and education level. Women were HPV-vaccinated with at least 1 dose.

In the analysis of attendance to two consecutive rounds, repeated attendance in HPV-vaccinated women of missing or lower education was not higher than in unvaccinated women of the same educational level (HR_adj_ 1.35, 95% CI 0.51–3.61 and HR_adj_ 1.09, 95% CI 0.75–1.57, respectively). However, repeated attendance was higher in HPV-vaccinated women with high school or university education (HR_adj_ 1.14, 95% CI 1.03–1.25 and HR_adj_ 1.14, 95% CI 1.08–1.19, respectively) than in women with high school or university education that were not vaccinated (S1 Table).

### Sensitivity analyses

When defining as HPV vaccinated only those vaccinated with the full 3 doses, results remained unchanged (S2 Table). Alternate time definitions of the first screening round did not change the results (S3 Table).

When including calendar year as a covariate, the positive association between HPV vaccination and screening attendance remained unchanged (HRadj 1.18, 95% CI 1.14–1.22).

## Discussion

In this nationwide study, we found that opportunistic HPV-vaccination is associated with an equal or higher attendance after invitation to cervical screening in Sweden. HPV-vaccinated women were also over-represented in the group that obtained a cervical smear without prior invitation. The overall association appeared mainly driven by the higher socioeconomic status of those vaccinated, although our findings by rounds, or in repeated screening, did not appear entirely explained by such factors.

The strengths of this observational study derive from the quality of data included: we had complete national follow-up of all invitations sent, all organized and opportunistic smears taken, and the ability to individually link this to registers of socioeconomic status and HPV vaccination status. This enabled a thorough evaluation of potential confounding and effect-modifying factors, as well as sensitivity analyses. The internal validity of our study should thus be high. The main limitation of this study is that women were not randomized to HPV vaccination, but self-selected whether to vaccinate or not and the women who did so constituted a minority of the study cohort (around 4900 women out of around 630 000). For women in this study, the cost of vaccination was unsubsidized due to their age being over 17 and those who got vaccinated would have done so at their own (or a parent’s) expense. Therefore, socioeconomic selection mechanisms were involved, and the relatively few women who actively chose to get HPV-vaccinated might also be more health-conscious, and prone to attend cervical screening from the outset. We have addressed this self-selection to HPV-vaccination in several ways. Firstly, adjustments were made for education level to account for the unequal uptake of vaccination across different levels of education.

Furthermore, the study population was restricted to those individuals who were invited to cervical screening as women that spontaneously/opportunistically obtain a cytological smear might be even more health conscious and thus less representative. Furthermore, it should be noted that the vaccine register SVEVAC had an estimated registration coverage of 80–85% during some of the years under study, for women that were vaccinated outside the subsidized vaccination program. This may have resulted in misclassification of the exposure where some HPV-vaccinated women were classified as unvaccinated. Since we found that HPV-vaccinated women were generally more likely to attend screening as compared to unvaccinated women, the effects of such misclassification would have been to slightly increase the screening rate in the comparison group of women classified as unvaccinated. This could mean that the observed positive association between HPV vaccination and screening attendance is somewhat underestimated in size, due to bias towards the null. However, since HPV-vaccinated women were few compared to unvaccinated, such misclassification should be unlikely to have substantially biased our results.

We emphasize that these women are not representative of the birth cohorts who are today being HPV-vaccinated as girls age 10–17 years old, nor of women vaccinated through organized programs. Therefore, in terms of external validity, our findings are likely best compared to other opportunistically vaccinated cohorts. It will be equally important to monitor future screening participation in those Swedish females who are now being vaccinated for free as school girls, who may differ in their attitude towards, and understanding of, screening than those vaccinated at a more mature age. Indeed, the Public Health Agency of Sweden is planning for future and regular follow-up of screening rates in HPV-vaccinated girls, to maintain vigilance for the possibility of changing behaviour over time. However, it is still important to perform early studies, such as the present work, of women that are HPV vaccinated, should the outcome have been the reverse and screening attendance reduced.

To our knowledge, this is the first nation-wide study to include individual-level data from the whole population and focus on cervical screening attendance after HPV vaccination. To our knowledge, this is also the first study to fully adjust for socioeconomic confounding, report response to invitations, and attendance to a second round of screening. Particularly the latter should be of value, since it is conceivable that HPV-vaccinated women duly attend the first round after vaccination, but then reduce their attendance in the second round due to the double perception of protection from both vaccination and preceding screening. However, this was not the case in our findings.

Although there have been theoretical studies on attitudes to screening after vaccination, [11–14, 20] these have shown conflicting findings depending on age group and education level. Studies that analyze actual participation have so far been relatively few, since in most countries HPV-vaccinated cohorts have not reached screening ages. Those studies that exist are mainly in line with our results. In Wales, young women are currently invited to cervical screening already at 18 years of age and a recent study from this area showed that screening participation was higher in HPV-vaccinated females, although in absolute terms screening participation was still generally low at only around 55% [21]. Similarly, a Danish study investigating girls born 1989–1999 showed that crude opportunistic screening participation was higher in HPV-vaccinated girls in the age group below ours, although the work focused on vaccine effectiveness and thus did not elaborate on these analyses [22]. A recent US study using national survey sample data with a response rate of around 61–63% also reported a positive association between HPV vaccine initiation in young women and having recently been screened [23].

In contrast to their and our findings, though, an Australian study on women vaccinated within the organized program in the region of Victoria recently reported that HPV-vaccinated women age 20–34 had significantly lower rates of 3-year screening attendance, although limitations to the ability of linking registers on the individual level in the study precluded determination of the exact size of the reduction in screening [24].

These data clearly further illustrate the need for comprehensive assessment of screening attendance in different vaccination settings. Possible reasons for the contrast in our results as compared to the Australian study include differences in whether women were vaccinated in an organized or opportunistic setting; how the invitation system to screening is designed, and ongoing background trends in screening participation in general. Indeed, Australia is observing a declining trend in participation among women [24] whereas this is not the case in Sweden. There was previously a declining trend in screening coverage for the youngest Swedish women invited, i.e. those 23–25 years, but since 2006, coinciding with the introduction of HPV vaccination in the nation, coverage in this age group has increased considerably, from ~67% to ~87% [6]. However, in the age group 26–30, overall coverage is still at around 75%, i.e. below the previous recommendation of 85% [6]. This is also evident from the long-term attendance presented here for unvaccinated women, meaning that continued attention is needed regarding screening practices in young adult women. And although the findings in the current study are reassuring, we propose that the follow-up of screening participation among HPV-vaccinated women be included as a new metric in audits of screening coverage wherever possible. This will allow early detection of any negative trends that might appear as we move into cohorts vaccinated at younger ages.

Our findings also imply that studies should take increased screening rates in vaccinated women into consideration when assessing the effectiveness against screen-detected cervical cancer precursor lesions, especially in those vaccinated after sexual debut where effectiveness may be compromised. If HPV-vaccinated women participate more extensively in screening, the recorded incidence of screen-detected lesions may be paradoxically somewhat increased, compared to the recorded incidence in unvaccinated—and less frequently screened—women.

This potential for detection bias may lead to an underestimation of vaccine effectiveness against cervical lesions in population-based studies of screened populations only, unless the potentially differential attendance rates are adjusted for.

In conclusion, opportunistic HPV vaccination was associated with an equal or higher attendance to cervical screening in Sweden in the first wave of self-selected, early adopters of the vaccine. This finding may be of relevance when assessing population-based effectiveness of opportunistic HPV vaccination against screen-detected cervical lesions. Continued regular follow-up of cohorts vaccinated in the free of charge, organized vaccination program is necessary, and regardless of vaccination status, all women will benefit from collective efforts to maintain high fidelity to screening programs.

## Supporting Information

S1 Fig Flow chart over the women not invited to screening during the study period, by explanation. (TIF)

S2 Fig Screening attendance after date of invitation by vaccination status and education level. (EPS)

S1 Table Adjusted hazard ratios of screening attendance to two consecutive screening rounds in HPV-vaccinated women compared to unvaccinated women. (DOCX)

S2 Table Adjusted hazard ratios of screening attendance in HPV-vaccinated women compared to unvaccinated women during the entire study period, and by round 1 and 2 during follow-up. (DOCX)

S3 Table Adjusted hazard ratios of screening attendance in HPV-vaccinated women compared to unvaccinated women for screening round 1 using various time cut-offs. (DOCX)
