# Supplementary figures and images for: The Participation of HPV-Vaccinated Women in a National Cervical Screening Program: Population-Based Cohort Study
Source: PLoS One. 2015 Jul 28;10(7):e0134185. doi: 10.1371/journal.pone.0134185 (PMC4517931; doi:10.1371/journal.pone.0134185)

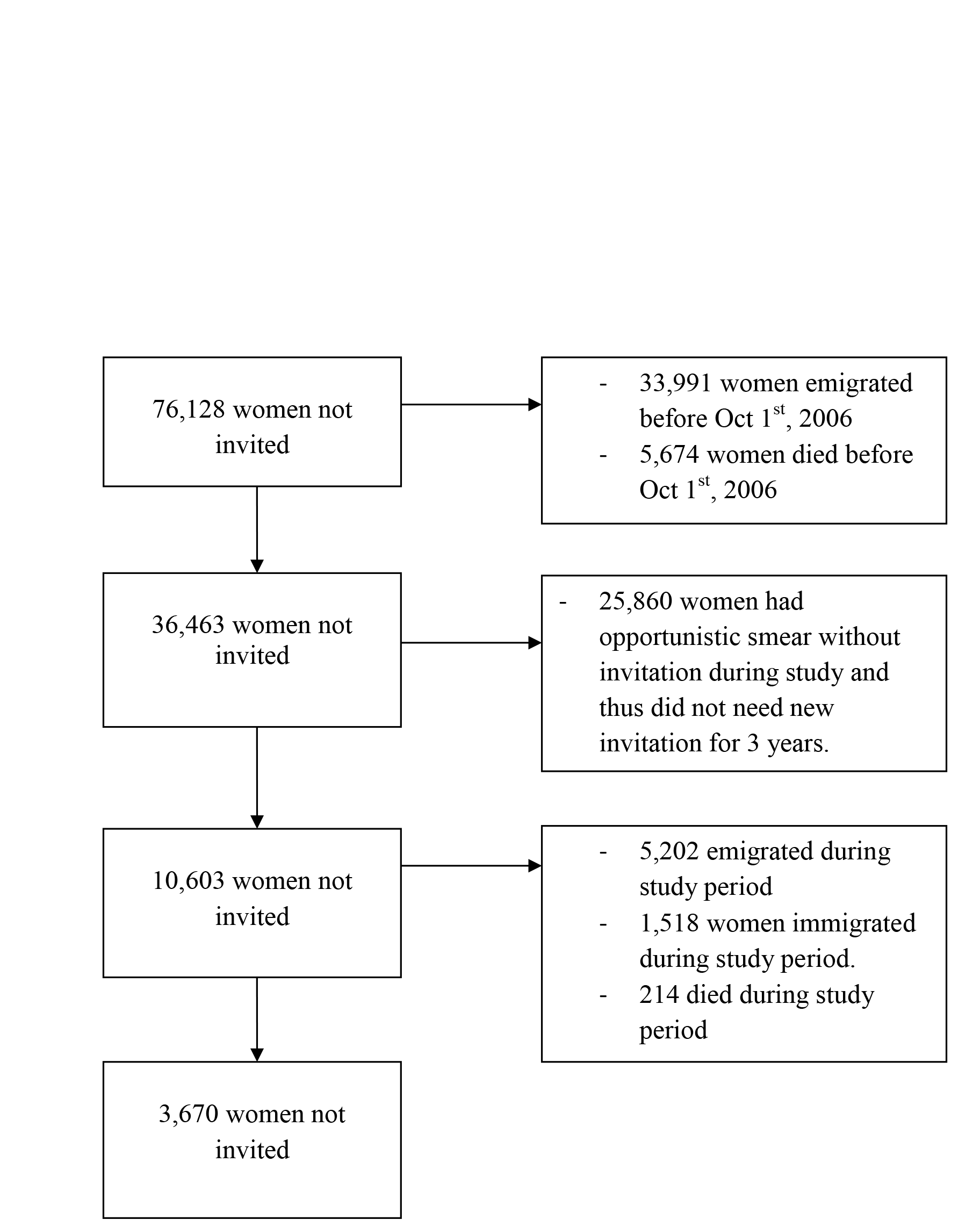

Supplement: S1 Fig — (TIF) [file pone.0134185.s001.tif]
