# Supplementary material for: The Participation of HPV-Vaccinated Women in a National Cervical Screening Program: Population-Based Cohort Study
Source: PLoS One. 2015 Jul 28;10(7):e0134185. doi: 10.1371/journal.pone.0134185 (PMC4517931; doi:10.1371/journal.pone.0134185)
Supplement: S1 Table — (DOCX) [file pone.0134185.s003.docx]

**S1 Table. Adjusted hazard ratios of screening attendance to two consecutive screening rounds in HPV-vaccinated women compared to unvaccinated women.**

|  | Attendance to two consecutive rounds | |
| --- | --- | --- |
|  | HR_adj_ ≥1 dose  (95% CI)^a^ | *P*  value |
| Missing data on education |  |  |
| Unvaccinated | Ref. |  |
| Vaccinated | 1.35 (0.51-3.61) | *0.545* |
| < High school |  |  |
| Unvaccinated | Ref. |  |
| Vaccinated | 1.09 (0.75-1.57) | *0.666* |
| High school |  |  |
| Unvaccinated | Ref. |  |
| Vaccinated | 1.14 (1.03-1.25) | *0.012* |
| University studies |  |  |
| Unvaccinated | Ref. |  |
| Vaccinated | 1.14 (1.08-1.19) | *<0.001* |

^a^ Hazard ratios (HRs) with corresponding confidence intervals (CIs) adjusted for income and including an interaction term between vaccination and education level. Women were HPV-vaccinated with at least 1 dose.
