# Supplementary material for: The Participation of HPV-Vaccinated Women in a National Cervical Screening Program: Population-Based Cohort Study
Source: PLoS One. 2015 Jul 28;10(7):e0134185. doi: 10.1371/journal.pone.0134185 (PMC4517931; doi:10.1371/journal.pone.0134185)
Supplement: S2 Table — (DOCX) [file pone.0134185.s004.docx]

**S2 Table. Adjusted hazard ratios of screening attendance in HPV-vaccinated women compared to unvaccinated women during the entire study period, and by round 1 and 2 during follow-up.**

|  | Attendance to screening over entire study period | | Attendance to  screening round 1 | | Attendance to  screening round 2 | |
| --- | --- | --- | --- | --- | --- | --- |
|  | HR_adj_ 3 doses^a^  (95% CI) | *P*  value | HR_adj_ 3 doses^a^  (95% CI) | *P*  value | HR_adj_ 3 doses^a^  (95% CI) | *P*  value |
| Unvaccinated | Ref. |  | Ref. |  | Ref. |  |
| HPV-vaccinated^b^ | 1.06 (1.02-1.10) | *0.005* | 1.10 (1.05-1.14) | *<0.001* | 1.20 (1.14-1.26) | *<0.001* |
|  |  |  |  |  |  |  |
| *By education level*^c^ |  |  |  |  |  |  |
| Missing data on education |  |  |  |  |  |  |
| Unvaccinated | Ref. |  | Ref. |  | Ref. |  |
| Vaccinated | 0.85 (0.49-1.51) | *0.587* | 1.08 (0.60-1.95) | *0.793* | 2.09 (0.67-6.48) | *0.203* |
| < High school |  |  |  |  |  |  |
| Unvaccinated | Ref. |  | Ref. |  | Ref. |  |
| Vaccinated | 1.16 (0.84-1.60) | *0.364* | 1.30 (0.94-1.81) | *0.111* | 1.26 (0.75-2.13) | *0.388* |
| High school |  |  |  |  |  |  |
| Unvaccinated | Ref. |  | Ref. |  | Ref. |  |
| Vaccinated | 1.14 (1.07-1.22) | *<0.001* | 1.16 (1.09-1.24) | *<0.001* | 1.23 (1.10-1.38) | *<0.001* |
| University studies |  |  |  |  |  |  |
| Unvaccinated | Ref. |  | Ref. |  | Ref. |  |
| Vaccinated | 1.01 (0.96-1.06) | *0.625* | 1.06 (1.00-1.11) | *0.038* | 1.19 (1.12-1.26) | *<0.001* |

^a^ Women were HPV-vaccinated with full 3 doses.

^b^ Hazard ratios (HRs) with corresponding confidence intervals (CIs) were adjusted for income and education level.

^c^ HRs with corresponding CIs were adjusted for income and including an interaction term between vaccination and education level.
