# Supplementary material for: The Participation of HPV-Vaccinated Women in a National Cervical Screening Program: Population-Based Cohort Study
Source: PLoS One. 2015 Jul 28;10(7):e0134185. doi: 10.1371/journal.pone.0134185 (PMC4517931; doi:10.1371/journal.pone.0134185)
Supplement: S3 Table — (DOCX) [file pone.0134185.s005.docx]

**S3 Table. Adjusted hazard ratios of screening attendance in HPV-vaccinated women compared to unvaccinated women for screening round 1 using various time cut-offs.**

|  | Attendance to screening round 1 | | | | | |
| --- | --- | --- | --- | --- | --- | --- |
|  | 2.5 year cut-off | | 3.5 year cut-off | | 4 year cut-off | |
|  | HR_adj_ 3 doses^a^  (95% CI) | *P*  value | HR_adj_ 3 doses^a^  (95% CI) | *P*  value | HR_adj_ 3 doses^a^  (95% CI) | *P*  value |
| Unvaccinated | Ref. |  | Ref. |  | Ref. |  |
| HPV-vaccinated | 1.11 (1.07-1.14) | *<0.001* | 1.08 (1.05-1.12) | *<0.001* | 1.07 (1.04-1.11) | *<0.001* |

^a^ Hazard ratios (HRs) with corresponding confidence intervals (CIs) adjusted for income and education level. Women were HPV-vaccinated with 3 doses.
